# An updated pharmacological insight into calotropin as a potential therapeutic agent in cancer

**DOI:** 10.3389/fphar.2023.1160616

**Published:** 2023-04-17

**Authors:** Jovana Rajkovic, Radmila Novakovic, Jelica Grujic-Milanovic, Alibek Ydyrys, Nurzhanat Ablaikhanova, Daniela Calina, Javad Sharifi-Rad, Basem Al-Omari

**Affiliations:** ^1^ Institute for Pharmacology, Clinical Pharmacology and Toxicology, Faculty of Medicine, University of Belgrade, Belgrade, Serbia; ^2^ Institute for Medical Research, National Institute of the Republic of Serbia, University of Belgrade, Belgrade, Serbia; ^3^ Biomedical Research Centre, Al-Farabi Kazakh National University, Almaty, Kazakhstan; ^4^ Department of Biophysics, Biomedicine and Neuroscience, Al-Farabi Kazakh National University, Almaty, Kazakhstan; ^5^ Department of Clinical Pharmacy, University of Medicine and Pharmacy of Craiova, Craiova, Romania; ^6^ Facultad de Medicina, Universidad del Azuay, Cuenca, Ecuador; ^7^ Department of Epidemiology and Population Health, College of Medicine and Health Sciences, Khalifa University, Abu Dhabi, United Arab Emirates

**Keywords:** calotropin, anticancer, molecular targets, signaling pathways, apoptosis, cytotoxicity

## Abstract

Calotropin is a pharmacologically active compound isolated from milkweed plants like *Calotropis procera, Calotropis gigantea,* and *Asclepias currasavica* that belong to the Asclepiadaceae family. All of these plants are recognised as medical traditional plants used in Asian countries. Calotropin is identified as a highly potent cardenolide that has a similar chemical structure to cardiac glycosides (such as digoxin and digitoxin). During the last few years, cytotoxic and antitumor effects of cardenolides glycosides have been reported more frequently. Among cardenolides, calotropin is identified as the most promising agent. In this updated and comprehensive review, we aimed to analyze and discuss the specific mechanisms and molecular targets of calotropin in cancer treatment to open new perspectives for the adjuvant treatment of different types of cancer. The effects of calotropin on cancer have been extensively studied in preclinical pharmacological studies *in vitro* using cancer cell lines and *in vivo* in experimental animal models that have targeted antitumor mechanisms and anticancer signaling pathways. The analyzed information from the specialized literature was obtained from scientific databases until December 2022, mainly from PubMed/MedLine, Google Scholar, Scopus, Web of Science, and Science Direct databases using specific MeSH search terms. The results of our analysis demonstrate that calotropin can be a potential chemotherapeutic/chemopreventive adjunctive agent in cancer pharmacotherapeutic management.

## 1 Introduction

Cancer is a process that occurs in different cells of the body, resulting from acquiring genetic and epigenetic changes in them, which leads to uncontrolled cell growth (Lee and Kim, 2022; GBD 2019 Colorectal Cancer Collaborators, 2022). In tumor formation increased attendance of reactive oxygen species (ROS) is involved because they activate various oncogenic signaling pathways, induce mutation of DNA, and induce immune escape, which, together with the microenvironment, angiogenesis, often lead to metastasis (Mitroi et al., 2019; Tsoukalas et al., 2019; Nakamura and Takada, 2021; Scheau et al., 2021) Cancer drug treatments differ depending on the type and stage of the tumor (Buga et al., 2019). Due to the heterogeneity of cancers and their responses to treatment, major efforts are being made to improve existing therapeutic protocols or to find new adequate therapeutics (Ianoși et al., 2019; Iordache et al., 2019) In light of this, is still actual to explore natural products to develop the final anticancer drug entity, which has fewer side effects, is low-cost, and has better application. Numerous studies have reported that many plant species and their bioactive compounds have anti-cancer properties (Khan et al., 2022). The cytotoxic effects of different plant extracts are well known and many compounds isolated from the plants are being detailed and evaluated for anticancer activities in different *in vitro* and *in vivo* models. Solowey et al. (2014) investigated 17 whole plant extracts on ten different human cancer cells. The efficacy of cell death in tumor cells varied depending on the content of multiple molecules with antitumor activities but was often aligned with ethnobotanical sources of historical use (Solowey et al., 2014). Calotropin belongs to the cardenolides, which is a class of cardiac glycosides (Mo et al., 2016) (Supplementary File S1). Cardiac glycosides are prominent in the treatment of cardiovascular diseases treat as congestive heart failure in humans (Silva et al., 2018), but also as a poison in the African dart arrow (Agrawal et al., 2012). The main and first established mechanism of action of cardenolides such as calotropin was the inhibition of the sodium-potassium exchanger, Na^+^/K^+^-ATPase. This enzyme allows the active transport of Na^+^ and K^+^ ions through the cell membrane. It was observed that calotropin has a more perceptible impact on the myocardium compared to it on skeletal muscles, as myocytes have more active Na^+^/K^+^-ATPase (Koch et al., 2020). As consequence, this can allow for higher cardiac output by the cardiac muscles but can also lead to arrhythmia (Hoyer et al., 2011). In the upcoming years after calotropin isolation, many studies have been dedicated to its crystal and molecular structure, and new methods of isolation. In recent years, researchers have conducted numerous studies on the anticancer effects of calotropin. One of the reasons is the fact that cardenolides target the enzyme Na^+^/K^+^-ATPase and lead to dysregulation of the sodium-potassium ion gradients and disrupt the membrane potential (Vila Petroff et al., 2003). Others have sought to exhibit *in vitro* cytotoxic and cytotoxic effects of the cardiac glycosides against lung cancer (Ibrahim et al., 2014; Kim et al., 2016), and leukemia (Nakano et al., 2020). In preclinical models of the STK11 mutant lung cancer cells, the cardiac glycosides directly inhibit ATP function. Clinical-relevant doses of cardenolide inhibit cell proliferation and migration of these tumor cells (Kim et al., 2016). In leukemia, overexpression of MYC protein initiates proliferation and blocks cell differentiation, thus treatment of these cells with cardiac glycoside leads to inhibition of the MYC pathway (Da Costa et al., 2018). Pederson et al. (2020) reported that cardenolide display potency against two triple-negative breast cancer (TNBC) cells BT-549 and Hs578T cells. A similar study was done by Zhou et al. (2019) about the anticancer activity of colorectal cancer cells. Today, despite considerable efforts to find adequate anticancer therapy, cancer remains one of the leading causes of death worldwide (Nakamura and Takada, 2021). The use of traditional chemotherapy and radiation therapy in combination with surgery, and/or immunotherapies, and hormone therapy sometimes do not give satisfactory results, and therefore use of targeted therapies has become more common in recent times (Nakamura and Takada, 2021). The current review represents an update of the anticancer mechanisms, molecular targets, and signaling pathways of calotropin, a natural bioactive compound of current interest in the pharmacotherapeutic management of cancer.

## 2 Review methodology

To get a comprehensive overview of the most recent data on anticancer molecular mechanisms of calotropin, the following specialized databases were searched, such as PubMed/MedLine, Google Scholar, Scopus, Web of Science, and Science Direct using the following terms MeSH alone or in combination: “Antineoplastic Agents,” “Phytogenic/pharmacology,” “Apoptosis/drug effects,” “Calotropis/chemistry,” “Calotropis/metabolism,” “Cardenolides/chemistry”, “Cardenolides/isolation and purification”, “Cardenolides/pharmacology”, “Cell Line, Tumor, Cell Proliferation/drug effects”, “Reactive Oxygen Species/metabolism”, “Humans”, “Signal Transduction/drug effects”, “Reactive Oxygen Species/metabolism”. This study included articles published in English that proved the anticancer molecular mechanisms of calotropin. Pharmacological studies that did not specify anticancer mechanisms and specific signaling pathways, doses used in the experiment, and *in silico* studies without evidence from preclinical pharmacological studies were excluded. The most important data were summarized in tables and figures. The taxonomy of plant species was validated according to World Flora Online and the chemical structures according to PubChem (WFO, 2021; PubChem, 2022).

## 3 Natural sources, traditional and current uses

Calotropin (also known as Pecilocerin A, Pekilocerin A) primarily was isolated from *Calotropis procera* (Ait) R. Br., *Calotropis gigantea* (L.) R. Br (Cg) and *Asclepias subulata*, the plants belonging to the family Asclepiadaceae (Agrawal et al., 2012; Kadiyala et al., 2013; Rascón Valenzuela et al., 2015a). Plants from the family *Asclepiadoideae,* have been found in Africa, Asia, Europe, Australia, South America, and the tropics of North America. *Calotropis sp*. is common in Africa and Asia and widely distributed throughout India, Sri Lanka, Nepal, Maldives, South China and Malaysia, and Pakistan (Kadiyala et al., 2013). Other species of this family such as *Asclepias sp*., mostly known as the desert plants, are native to America (Rascón Valenzuela et al., 2015b). The mentioned species of the family *Asclepiadoideae* have been since ancient times part of traditional medicine for the treatment of various diseases related to the central nervous system, skin diseases, digestive system, respiratory system, reproductive system pain, and cancer (Rascón Valenzuela et al., 2015a). Different parts of these plants have been used since antiquity as a galenical in traditional medicine. Calotropin is isolated from latex, leaves, and root bark from plants (Kadiyala et al., 2013). Accidental poisoning is common in animals who have ingested milkweed. Interestingly, some insects species, the pyrgomorphid grasshopper *P. bufonius* and caterpillar of monarch butterflies eat milkweeds of the plants family *Asclepiadoideae* and stored calotropin as a defense mechanism against predators and parasites (Agrawal et al., 2012). Phytochemical studies of plants from family *Asclepiadoideae* have revealed the presence of several types of compounds such as cardenolide, pregnane glycosides, secopregnane glycosides, triterpenoids, steroidal glycosides (Elgamal et al., 1999). A native plant of the family *Asclepiadaceae* is distributed in the tropical and subtropical regions of the planet. In some of these regions, the dependence on traditional plant-based medicines persists until today, especially if you keep in mind that many parts of this plant are useable. In the past, these plants have been popular to cure several human diseases such as fever, asthma, rheumatism, indigestion, diarrhoea, and dysentery (Al-Taweel et al., 2017). Despite long evidence of using traditional plants that contain calotpropin as a part of folk medicine, its antitumor and cytotoxic effects have been examined just recently in a controlled laboratory condition. The first evidence of the cytotoxic effect of calotropin may be found in an article written by Kupchan et al. (1964) dated 1964. An alcoholic extract of *Asclepias curassavica* was tested on *in vitro* cells cultured from human carcinoma of the nasopharynx and the observed cytotoxic activity was attributed to calotropin isolated from that extract. The flower and leaf are widely used parts of *Calotropis procera (Cp)*. The leaf extract of *Cp* possessed a powerful antiulcer activity by to the reduction gastric acid secretion, as well as augmenting the mucosal defensive factors and inhibiting lipid peroxide levels (Al-Taweel et al., 2017). In a previous study, it has been shown that extracts from all parts of these plants have a variety of biological activities (Huang et al., 2018; Mutiah et al., 2018). The pharmaceutical potential of extracts gained from the bark and leaves of plant *Cp* showed notable antibacterial potential against different bacterial strains (Sharma et al., 2015; Pattnaik et al., 2017). The ethanol extract of the *Cp* in a dosage of 500 mg/kg induced significant antipyretic and analgesic activity in mice (Mossa et al., 1991). The study of Magalhães et al. (2010) reported that among five extracts, ethyl acetate and acetone extract of *Cp* displayed higher cytotoxic potential against two different tumor cells: colon (HCT-8) and melanoma (B-16) cells. The same authors also reported that those extracts induced a significant reduction in tumor weight as well as the growth of the sarcoma tumor in mice. Several previous studies of the biological activities of *Cp* have shown anti-inflammatory (Kumar et al., 2015; Kumar et al., 2022), and antifungal activity (Al-Rowaily et al., 2020; Freitas et al., 2020) were shown.

## 4 Therapeutic activity, cellular and molecular mechanisms of calotropin in various types of cancer

### 4.1 Brain cancer

A recent study investigated several types of cardiac glycosides isolated from *Calotropis gigantea* and showed that calotropin had the most pronounced cytotoxicity on A172 and U251 glioblastoma cell lines, and confirmed a possible anticancer mechanism that includes G2/M phase cell arrest (He et al., 2021). Another evidence that calotropin may have cytotoxic effects on cancer cell lines derives from its capability to inhibit the enzyme Na^+^/K^+^-ATPase. Na^+^/K^+^-ATPase is an important transmembrane protein found in all mammalian cells with the main role in cell ion homeostasis. A potential target for anticancer therapy contributes to its role in signal transduction, and overexpression, as well as in ion homeostasis, in severe neoplasms (Bejček et al., 2021). In research that was done by Meneses-Sagrero et al. (2022) anticancer effects of calotropin were analysed using commercial brain porcine Na^+^/K^+^-ATPase. Calotropin showed dose-response inhibitory effects on Na^+^/K^+^-ATPase activity (IC_50_ = 0.27 ± 0.06 μM). The IC_50_ value was by a previous study where was observed that calotropin has the same IC_50_ value on porcine cerebral cortex Na^+^/K^+^-ATPase activity (Agrawal et al., 2021). This value of IC_50_ of calotropin is very similar to other well-known cardenolides like ouabain and digoxin which indicated the great inhibitory potential of calotropin on Na^+^/K^+^-ATPase. Also, kinetic analysis on Na^+^/K^+^-ATPase activity showed that the nature of this inhibition is non-competitive (Meneses-Sagrero et al., 2022).

### 4.2 Breast cancer

**FIGURE 1:**
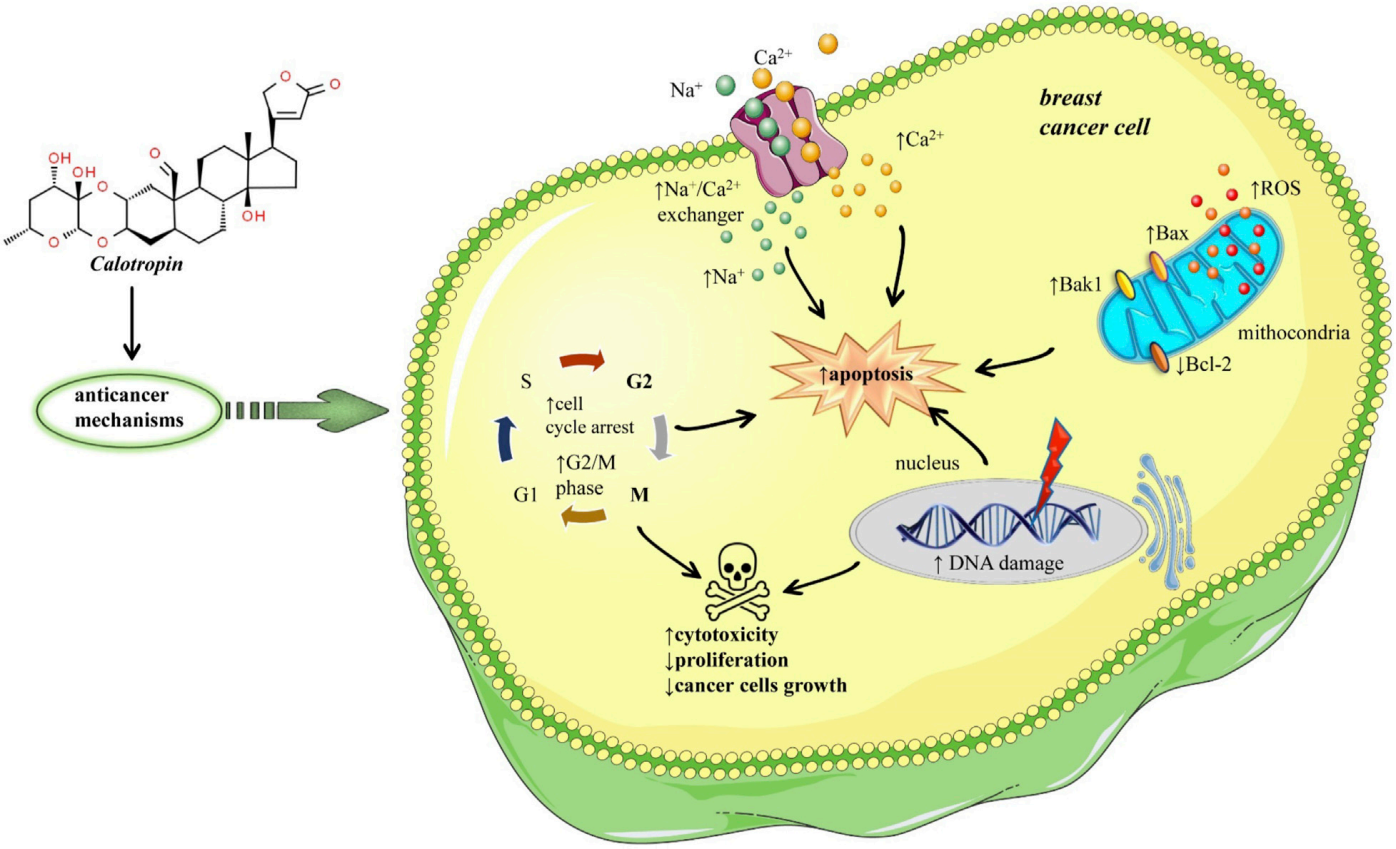
Potential mechanisms of anticancer effects of calotropin in breast malignant tumor. Calotropin inhibits Ca2+ efflux via the Na+/Ca2+ exchanger, increasing intracellular Na+ and Ca2+, triggering apoptosis. Calotropin also induces apoptosis and cell death in tumour cells by disintegrating DNA and arresting the cell cycle in the G2/M phase. It also increases Bax/Bak1 expression and significantly reduces Bcl-2 expression, so this Bax/Bcl-2 ratio plays an important role in determining apoptosis. Symbols: ↑increase, ↓decrease.

Breast cancer occurs when normal cells in the breast change and grow out of control and it occurs with a frequency of 13% in the global population, and TNBC represents 10%–20% of all breast cancers (Yao et al., 2017). More than a decade ago six TNBC subtypes of breast cancer cells have been identified based on tumor gene expression analyses, one of them being BT-549 cells (Pederson et al., 2020). Nine cardenolides from *Calotropis gigantea* exhibited selective cytotoxic activities on TNBC, but calotropin was notably more selective for BT-549 cells (Pederson et al., 2020). Regarding human triple-negative breast cancers (TNBC) cell lines were shown effects of calotropin different IC_50_ values and selectivity across different human TNBC cell lines (Pederson et al., 2020). Calotropin was more selective for BT-459 cells (IC_50_ = 0.03 ± 0.002 µM) and Hs578T cells (IC_50_ = 0.06 ± 0.01 µM) compared to MDA-MB-231 (IC_50_ value was 0.44 ± 0.08 µM). The results suggested that BT-459 cells are more sensitive to calotropin due to increases in intracellular Ca^2+^ levels, leading to cell death at lower concentrations than is required in other human TNBC cell lines. Another evidence that calotropin may have cytotoxic effects on cancer cell lines derives from its capability to inhibit the enzyme Na+/K + -ATPase viz altering through key transmembrane cellular protein homeostasis. The possible explanation of the different effects of calotropin in the different human triple-negative breast cancer cell lines may derive from the expression of different Na^+^/K^+^-ATPase isoforms and ↑Na^+^/Ca^2+^ exchanger (NCX) isoforms. It has been shown that α1 Na^+^/K^+^-ATPase isoform is more resistant to inhibition by some cardiac glycosides, and at the same time, its expression was lowest in BT-459 cells. However, in another cell line Hs578T the expression of α1 Na^+^/K^+^-ATPase isoform was intermediate. Related to another hypothesis with Na^+^/Ca^2+^ exchanger isoforms, it was observed that the mRNA of NCX1 isoform was significantly higher in BT-459 and Hs578T compared to the other human triple-negative breast cancer cell lines. Thus, the high mRNA expression of NCX1 may predict sensitivity to calotropin. The breast cancer BT-549 cells were treated with different doses of calotropin, 150 nM and 500 nM. The results showed that both doses of calotropin inhibit the efflux of Ca^2+^ via the Na^+^/Ca^2+^ exchanger. A major consequence of this inhibition is increased intracellular Na^+^, leading to increased Ca^2+^ entry and elevated concentrations of intracellular Ca^2+^. Cancer cells with defects in this signalling pathway might be particularly vulnerable, as an increase in intracellular Ca^2+^ triggers apoptosis (Vila Petroff et al., 2003) (Figure 1). Calotropin also induces apoptosis in breast cancer MCF-7 cell lines, by modulating the phosphatidylserine gene, DNA disintegration, and G2/cell cycle arrest. Also, this treatment increased the expression of Bax/Bak1 and significantly reduced Bcl-2 expression, so this ratio of Bax/Bcl-2 plays an important role in determining apoptosis (Kharat and Kharat, 2019) (Figure 1).

### 4.3 Lung cancer

**FIGURE 2:**
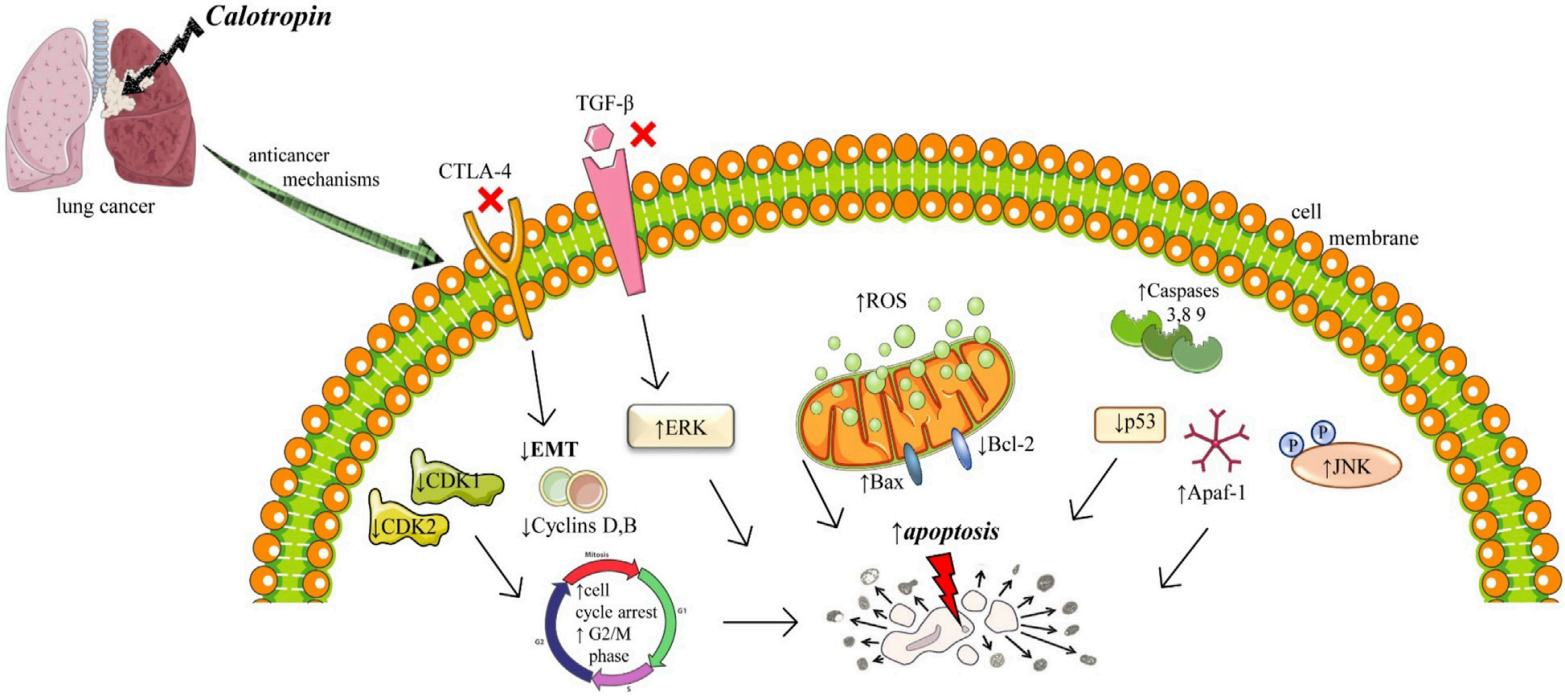
Illustrative scheme with cytotoxic mechanisms of Calotropin in lung cancer. Abbreviations and symbols: ↑ increase, ↓decrease, Epithelial-mesenchymal transition (EMT), Reactive oxygen species (ROS), mitogen-activated protein (MAP) kinases and cJun NH2-terminal kinase (JNK), transforming growth factor β (TGF-β), cytotoxic T-lymphocyte–associated antigen 4 (CTLA-4).

One of the most common types of human cancer is non-small cell lung cancer (NSCLC) which is characterized by invasion, migration, rapid growth, and reoccurrence (Guo et al., 2022). *In vitro* studies on NSCLC showed that treatment with calotropin promoted apoptosis by increasing the expression of pro-apoptosis genes: *caspase-3, caspase-8*, and apoptotic protease activating factor-1 (Apaf-1) and downregulated expression of anti-apoptotic proteins: p53, B-cell lymphoma (Bcl 2) and Bclw (Paesmans et al., 2015). On the same calotropin treatment of cancer cells activates the TGF-β/ERK signaling pathway, by decreasing the expression of TGF-β and ERK½, and downregulating phosphorylation of ERK½ (Paesmans et al., 2015). *In vivo* assay on NSCLC-bearing mice showed that calotropin treatment markedly inhibited tumor growth, increased the number of apoptotic cells, and significantly promoted apoptosis markers: caspase-3 and caspase-8. Long-term observation has shown that this treatment significantly prolonged the survival of these mice (Paesmans et al., 2015) (Figure 2).

Epithelial-mesenchymal transition (EMT) involves changes in the intracellular cytoskeleton and extracellular matrix. After EMT, cells lost polarization, acquire an elongated morphology, and possess strong intercellular adhesion and polarity, which can be attributed to the reorganization of the actin cytoskeleton as well as increased expression of vimentin or keratin (Grände et al., 2002). EMT is a complex which can be activated by different inflammatory stimuli like TGF-β growth factor, cytotoxic T-lymphocyte–associated antigen 4 (CTLA-4), hypoxia, or extracellular matrix components (Grände et al., 2002). CTLA-4 is a multifactorial protein with an important role in the communication process in cells, was significant downregulation in NSCLC cells after calotropin administration led to tumor inhibition. Also, calotropin inhibited the protein expression levels of TGF-β, an essential factor in carcinogenesis (Tian et al., 2018). Therefore, calotropin administration regulated apoptosis, inhibited tumor growth, and prolonged survival in NSCLC cells via the TGF-β/ERK signaling pathway (Tian et al., 2018).

Another research compared calotropin inhibitory and pro-apoptotic activity on two types of NSCLC cell lines: cisplatin-induced resistant NSCLC cell line (A549/CDDP) and its parent’s cells lung adenocarcinoma A549 (Mo et al., 2016). Interestingly, while A549/CDDP was significantly inhibited by calotropin in a concentration-dependent manner (IC_50_ 0.33 ± 0.06 μM at 48 h), the same inhibitory effect on A549 was weak (IC_50_ 38.46 ± 1.16 μM at 48 h). Furthermore, while cell cycle arrest in the G2/M phase and an increased number of cells in the apoptotic phase were observed in A549/CDDP after treatment with calotropin, the same effects lack in A549. After the treatment with calotropin the expression of CDK1, CDK2, cyclin A, and cyclin B was significantly downregulated in A549/CDDP cell line, while p21 and p53 protein levels were increased. The same as in the previously mentioned research, the expression levels of the precursor forms of Cap- 3, Cap-8, and Cap-9 were downregulated and their active forms were upregulated. Also, the increased intracellular levels of ROS were observed in the same cell lines, as well as changes in mitochondrial membrane potential and poly (ADP-ribose) polymerase (PARP) after treatment with calotropin (in a dose of 0.5 and 1.0 μM) compared to a control group. The levels of anti-apoptotic protein Bcl-2 were decreased and pro-apoptotic Bax protein levels were increased in a concentration-dependent manner in the treatment group. All of the previously mentioned results were not observed after the treatment of calotropin on the A549 cell line. In A549/CDDP cell line treatment with calotropin was correlated with increased phosphorylation of JNK (which has an essential role in signalling pathways of apoptosis), while this was not observed in the A549 cell line. There is no provided explanation of the differences in the effects of calotropin on these 2 cell lines (Mo et al., 2016).

The anticancer activity of calotropin on A549 cells through caspase-dependent apoptosis activated by extrinsic pathway was obtained in research done by Rascón-Valenzuela et al. (2016) and colleagues. A recent study (Rascón Valenzuela et al., 2015a) indicated that the IC_50_ values of calotropin on the same cell line are 0.0013 µM, while antiproliferative activity was confirmed on another two cancer cell lines LS 180 (human colorectal adenocarcinoma cell line) and PC-3 (human prostate cancer cell line) with IC_50_ values 0.06 and 0.41 µM, respectively. Also, the cytotoxic effects of calotropin on lung adenocarcinoma A549 cells were confirmed in the research done by Sweidan et al. (2021). Interestingly, they compared the effects of calotropin on human fibroblast cell lines as normal cells and the toxicity of calotropin was lower which indicated that calotropin is more selective to cancer cells compared to normal cells. The same observation on human normal cells was reported by Rascón-Valenzuela et al. (2015). The *in vitro* growth inhibitory activity of calotropin was tested on A549 cells, PC-3, and U373 (glioblastoma cell line) by Ibrahim and colleagues, and the same value of IC_50_ (0.005 µM) was reported for all 3 cell lines (Ibrahim et al., 2014).

Comprehensive research on the anticancer effects of calotropin was done by Tian et al. (2018). They used bronchioalveolar carcinoma H358 cells treated with calotropin (0.50 mg/mL) for 24 h, 48 h, and 72 h based on the subject of analysis to investigate the inhibitory effects of calotropin on the growth and aggressiveness of cancer cells. Also, *in vivo* study was performed using 80 specific pathogen-free female nude (6–8 weeks old, weight 30–35 g) mice. All of them were subcutaneously injected with H358 cells and after that divided into 2 groups. In the treatment group, mice were intravenously injected with calotropin (5.0 mg/kg) every day for 7 days, while PBS injections were used as the control. Related to *in vitro* experiments on H358 cells it was observed that calotropin suppressed cell growth in a time-dependent manner (after 72 h treatment). The cell proliferation was arrested at the G2/M phase. Also, calotropin inhibited protein level expression of cyclins (cyclin-dependent kinases: CDK1 and CDK2), fibronectin, vimentin and E-cadherin. The migration and invasion were inhibited after treatment with calotropin. Analysis of calotropin effects on H358 cells apoptosis showed that apoptosis was promoted. There was increased pro-apoptosis gene expression of caspase-3 (Cap-3), caspase-8 (Cap-8), and Apaf-1, while anti-apoptosis protein expression levels of p53, Bcl-2, and Bsl-w were decreased. Also, calotropin promoted Cyt-c and JNK protein expression levels. Additional effects of calotropin indicated that its treatment of H358 cells decreased the CTLA-4 (T-lymphocyte-associated-protein 4) protein expression (CTLA-4 protein is a checkpoint receptor that downregulates T cell activation), and inhibited the TGF-β protein expression levels and phosphorylation and protein expression levels of ERK1/2, indicated that probably TGF-β/ERK signalling pathway is included in calotropin-mediated apoptosis.

### 4.4 Digestive cancers

#### 4.4.1 Liver cancer

One of the most aggressive cancers with a poor prognosis is liver cancer, the most common type being hepatocellular carcinoma (HCC). The anticancer activity of calotropin in HCC was linked to the suppression of fatty acid levels by a decrease in inflammatory cytokines and adipocyte shrinkage, as can be seen in a different model of liver carcinogenesis (Zhang et al., 2020). During the early phase of hepatocarcinogenesis, inflammatory and parenchymal cells release large amounts of IL-6 and TNF-α which is responsible for the release of TGF-β1. The elevated level of inflammatory cytokines triggers mitogen-activated protein (MAP) kinases and cJun NH2-terminal kinase (JNK), which is implicated in the progression of liver fibrosis and deposition of collagen. Calotropin treatment decreases IL-6 and TNF-α expression, implying that the inflammatory process suppresses and slows the progression of carcinogenesis (Pan et al., 2018). Also, the IC_50_ value of calotropin was reported on the human hepatocarcinoma cell line (HepG2) and Raji cells (human B lymphoblastoid cell line), and values were 0.04 and 0.02 µM, respectively, which confirmed its cytotoxic effect on tumor cell lines (Li et al., 2009).

Recently, the pro-apoptotic effects of calotropin were confirmed on HepG2 and human colorectal adenocarcinoma cells (Caco-2) (Martucciello et al., 2022).

#### 4.4.2 Colorectal cancer

**FIGURE 3:**
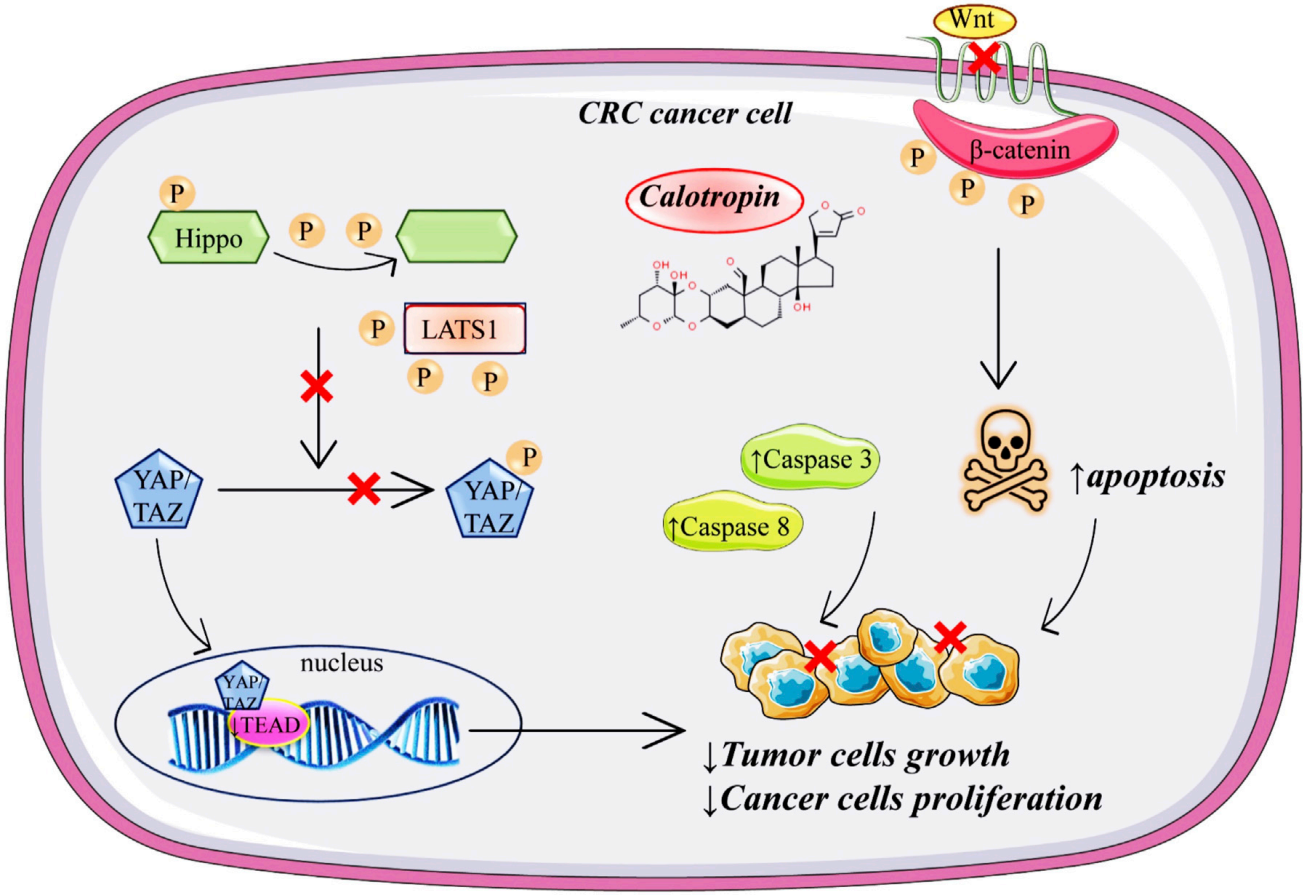
Schematic representation of anticancer mechanisms of calotropin in colorectal cancer.

Colorectal cancer (CRC) is a malignant neoplasm that develops in the colon and rectum, and its symptoms can be extremely varied; in its early stages, CRC is asymptomatic and is detected through screening examinations (2022). Calotropin markedly suppresses the proliferation of colorectal cancer cells by the Hippo pathway through the activity of YAP (Zhou et al., 2019). Since the Hippo pathway is a highly complex signaling network with >30 components, dysregulation of the Hippo pathway components often leads to aberrant cell growth and tumor formation (Meng et al., 2016). Treatment with calotropin induces YAP dephosphorylation in colorectal cancer cells. Dephosphorylation of YAP, after calotropin treatment, induced its translocation into the nucleus of the colorectal cancer cells. After nuclear localization, YAP interacts with the transcription factors of the TEAD family (Figure 3). Downregulation of the TEAD target gene, after administration of calotropin decrease the expansion of progenitor cells, and tissue overgrowth. Together, these data showed that calotropin inhibits tumor growth in colorectal cancer cells (Meng et al., 2016) (Figure 3). When *in vivo* experiments of a 25-day short-term observation period were analysed it was suggested that injection of calotropin inhibited tumor growth. In the treatment group, the number of apoptotic cells and lymphocyte infiltration was increased. In excised tumors from the treatment group expression levels of Casp-3 and Casp-8 were increased. Besides the fact that the administration of calotropin has significantly inhibited tumor growth, after the 120-day observation period the prolonged mice survival was noted as well.

In another research done by Zhou et al. (2019), it was indicated that calotropin may inhibit tumor growth. The *in vitro* study was done on two types of human colorectal cancer cell lines HT-29 and HCT116, treated with calotropin for 24 h. Increased concentrations of calotropin (0.2–10 µM) inhibit proliferation in a dose-dependent manner in both cancer cell lines. Also, calotropin promoted dephosphorylation of YAP (Yes-associated protein) and induced its nuclear localization in colorectal cancer cells, therefore supporting the hypothesis that the Hippo signalling pathway is included in calotropin-induced inhibition of cell proliferation. Additionally, calotropin treatment shortened the half-life of LATS1 (large tumor suppressor 1) protein, the main kinase in the Hippo pathway that phosphorylates YAP. Calotropin probably promotes LATS1 degradation through the ubiquitination/proteasome pathway. Another part of the study was *in vivo* performed with BALB/c immunodeficient nude mice injected with HT-29 cells (6–8 weeks old). Mice were divided into two groups, the treatment group where calotropin was intravenously administered once every 2 days for 3 weeks, and the control group was treated in the same way during the same period with DMSO as a vehicle. It has been shown that the tumor obtained from the calotropin-treatment group was reduced in volume and weight. Another research on colon cancer cells (SW480 colon adenocarcinoma cell line) marked calotropin as an anticancer agent which inhibits the Wnt signalling pathway by decreasing nuclear and cytosolic located β-catenin in a dose-dependent manner and leading to degradation of β-catenin by increasing the phosphorylation of β-catenin through casein kinase 1α (CK1α) (Park et al., 2014). Additionally, they concluded that calotropin increased mRNA and protein levels of CK1α. Further, since the Wnt signalling pathway is recognised as a regulator of the early and late stages of apoptosis, the early signs of apoptosis on the SW480 colon cancer cell line were observed after calotropin (5.2 nM) exposure for 24 h.

### 4.5 Leukemia

**FIGURE 4:**
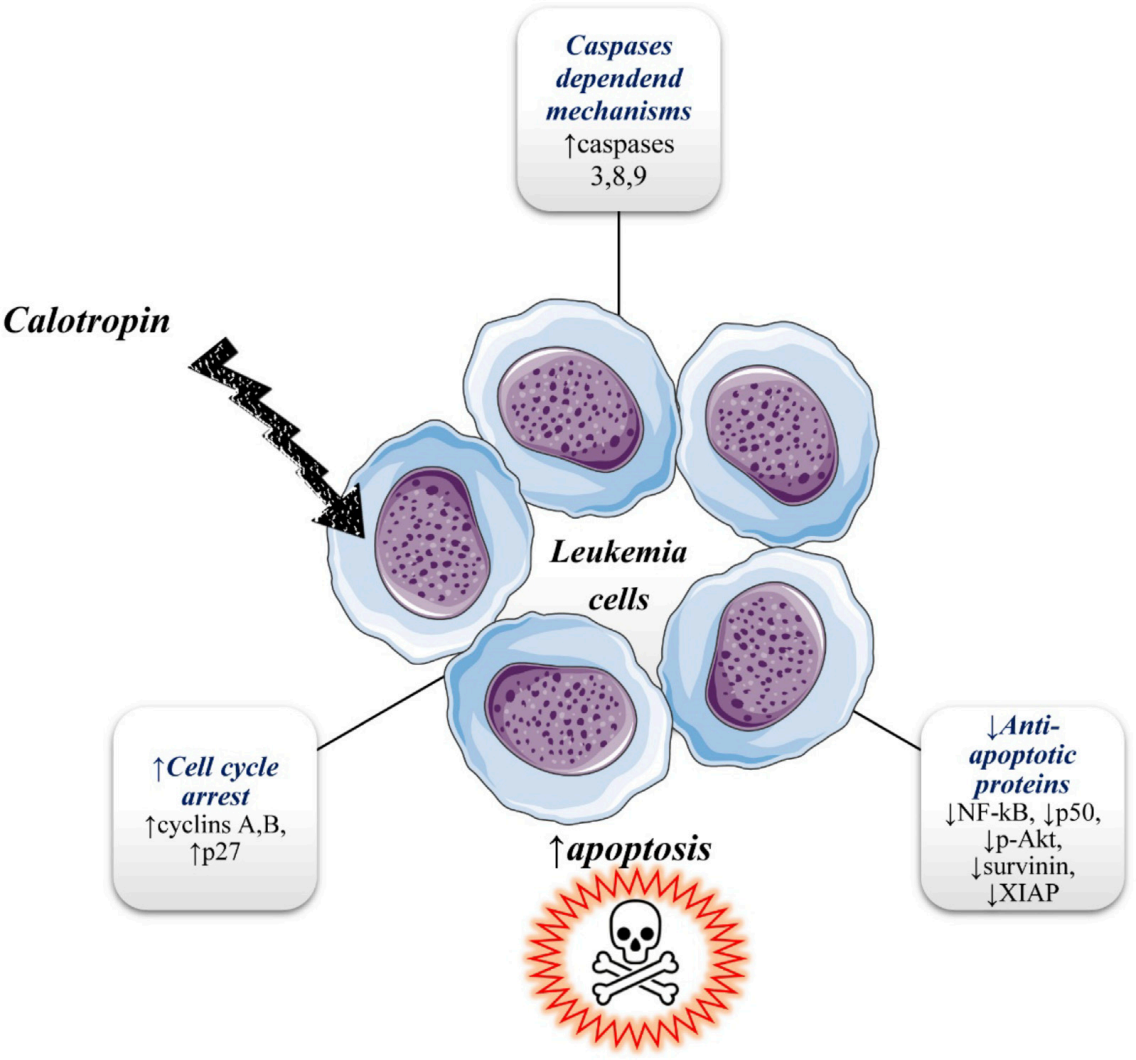
Illustrative diagram with anti-leukemic mechanisms of calotropin. Symbols: ↑increase, ↓decrease.

The observation that the anticancer effects of calotropin are probably mediated in a caspase-dependent manner was for the first time reported on human chronic myeloid leukemia K562 cells in research done by Wang et al. (2009). In the same research, calotropin treatment showed that the human acute myeloid leukemia HL 60 cells were more sensitive to cytotoxicity than K562 cells (K562 cells are the model system for the study of resistance to chemotherapy). The growth of cells was inhibited in a time and dose-dependent manner (0.01–20 μg/mL) in the G2/M phase with observed downregulation of cyclins, cyclin A, and cyclin B, and upregulation of p27 in K562 cells. It was suggested that calotropin may induce apoptosis through the caspase-dependent mechanism because in a dose-dependent manner was observed increases in the activity of Casp-3, Caps 8, and Casp-9 after calotropin treatment. Also, in a dose-dependent manner treatment with calotropin inhibit the expression of anti-apoptotic proteins and inhibitors of apoptotic proteins including NF-κB, p50, p-Akt and survivin, and XIAP (Figure 4). Calotropin is considered a potential agent for the treatment of adult T-cell leukemia/lymphoma (ATL). Nakano et al. (2020) showed that calotropin stopped the proliferation of MT-1 and MT-2 cells at the G2/M phase and promote apoptosis. At the same time, lower toxicity of calotropin was expressed toward normal PB-MNCs (peripheral blood mononuclear cells) compared to human T-cell leukemia virus type I infected T-cell lines (HTLV-I). MT-1 was derived from leukemia cells of peripheral blood from a patient with ATL, while the MT-2 cell line was derived from normal human cord leukocytes of the healthy donor which were co-cultivated with leukemia cells from an ATL patient (Yoshida et al., 1982). Table 1

**TABLE 1:** Summarized data obtained from preclinical pharmacological studies regarding calotropin anticancer activity.

Cancer type	Experimental model	Molecular mechanisms/signaling pathways	Results	Ref
***Brain cancer***	*in vitro*	↑cell arrest in G2/M phase	↑cytotoxicity	He et al. (2021)
	A172, U251 glioblastoma cells	↓Na^+^/K^+^-ATPase	IC_50_ = 0.27 ± 0.06 μM	
***Breast cancer***	*in vitro*	↑intracellular Ca^2+^	↑cytotoxicity	Pederson et al. (2020)
	BT-549, Hs578T	↓Na^+^/K^+^-ATPase	↑ apoptosis	
	MDA-MB-231	↓NCX1	IC_50_ = 0.03 ± 0.002 µM for BT-459	
	TNBC cells	↑intracellular Ca^2+^	IC_50_ = 0.06 ± 0.01 µM for Hs578T	
			IC_50_ = 0.44 ± 0.08 µM for MDA-MB-231	
	*in vitro*	↓Na^+^/K^+^-ATPase	↑ apoptosis	Vila Petroff et al. (2003)
	BT-549 cells	↑intracellular Ca^2+^	IC_50_ = 150–500 nM	
	*in vitro*	↑DNA disintegration	↑ apoptosis	Kharat and Kharat (2019)
	MCF-7 cells	↑G2/M cell cycle arrest	IC_50_ = 40 μg/mL	
		**↑**Bax/Bak1, ↓Bcl-2		
***Lung cancer***	*in vitro*	↑ caspases 3,8; ↑Apaf-1	↑ apoptosis	Paesmans et al. (2015)
	non-small cell lung cancer	↓p53, ↓Bcl2, ↓Bclw	↓tumor growth	
	NSCLC cells *in vivo*	↑TGF-β/ERK	↑apoptotic cells	
	NSCLC-bearing mice		↑survival of mice	
	*in vitro*	↑TGF-β/ERK	↓tumor growth	Tian et al. (2018)
	NSCLC cells		↓carcinogenesis	
	*in vitro*	↑cycle arrest in the G2/M phase	↑ apoptosis	Mo et al. (2016)
	A549/CDDP cells	↓CDK1, ↓CDK2	IC_50_ = 0.33 ± 0.06 μM for A549/CDDP cells	
	A549 cells	↓cyclin A, B	IC_50_ = 38.46 ± 1.16 µM for A549 cells	
		↑p2, ↑p53, ↓Cap- 3, 8, 9		
		↑ROS, ↑PARP, ↓Bcl-2, ↑Bax, ↑JNK		
	*in vitro*	↑caspases	↑ apoptosis	Rascón-Valenzuela et al. (2016)
	A549 cells		IC_50_ = 0.0013 µM	
	*in vitro*	↑G2/M cell cycle arrest	↓cancer cells growth	Tian et al. (2018)
	bronchioalveolar carcinoma	↓CDK1, ↓CDK2, ↓fibronectin, ↓vimentin, ↓E-cadherin	Dose = 5.0 mg/kg	
	H358 cells *in vivo*	↑caspases	↑ apoptosis	
	mice injected with H358 cells	↓p53, ↓Bcl-2, ↓Bsl-w	IC_50_ = 0.50 mg/mL	
		↓CTLA-4, ↓TGF-β/ERK		
***Liver cancer***	*in vitro*	↓IL-6, ↓TNF-α	↑cytotoxicity anti-inflammatory, ↓carcinogenesis	Li et al. (2009)
	hepatocarcinoma	↓TGF-β1, ↓JNK	IC_50_ = 0.04–0.02 µM	
	HepG2 cells			
***Colorectal cancer***	*in vitro*	↓Hippo pathway	↓proliferation	Meng et al. (2016)
	CRC cells *in vivo*	↑YAP dephosphorylation	↑apoptosis	
	mice with CRC	↓TEAD	↑lymphocyte infiltration	
		↑Cap-3, 8	↓ cancer cells growth	
			↓tumor formation	
			↑mice survival	
	*in vitro*	↑YAP dephosphorylation	↓cell proliferation	Zhou et al. (2019)
	HT-29, HCT116 cells *in vivo*	↓Hippo	↓tumor growth	
	BALB/c immunodeficient nude mice injected with HT-29 cells	↓LATS1	↓ tumor volume and weight	
			IC_50_ = 0.2–10 µM	
	*in vitro*	↓Wnt, ↓β-catenin	↑apoptosis	Park et al. (2014)
	SW480 cells	↓CK1α, ↑mRNA	IC_50_ = 5.2 nM	
***Leukemia***	*in vitro*	↑cell arrest in G2/M phase	↓ cancer cells growth	Wang et al. (2009)
	human chronic myeloid leukemia	↓cyclin A, B	↑apoptosis	
	K562 cells human acute myeloid leukemia HL 60 cells	↑Cap-3, 8,9, ↑p27	IC_50_ = 0.01–20 μg/mL	
		↓NFκB/p50, ↓p-Akt		
		↓survivin		
		↓XIAP		
	*in vitro*	↑cell arrest in G2/M phase	↑apoptosis	Nakano et al. (2020)
	adult T-cell leukemia/lymphoma ATL cells			

Abbreviations and symbols: ↑upregulated, ↓downregulated, B-cell lymphoma (Bcl 2), cisplatin-induced resistant NSCLC cell line (A549/CDDP), Yes-associated protein (YAP), large tumor suppressor 1 protein (LAST1), casein kinase 1α (CK1α), caspase (Cap), apoptotic protease activating factor-1 (Apaf-1), Na^+^-Ca^2+^ exchanger (NCX), bcl-2-like protein 4 (Bax), interleukin (IL), X-linked inhibitor of apoptosis protein (XIAP), transforming growth factor β (TGF-β), Nuclear factor kappa-light-chain-enhancer of activated B cells (NF-κB).

## 5 Calotropin synergistic effects with chemotherapeutic agents

Standard treatments currently involve chemotherapy with or without surgical resection but there are still certain limitations. Using traditional plant extracts as an alternative therapeutic strategy is subject to much research. Standard chemotherapy is associated with drug resistance and the occurrence of systemic adverse effects on different organs that limit its utility Therefore, to overcome these barriers and achieve better therapeutic success, there are recommendations in some studies of a combination regimen of traditional chemotherapeutic and medicinal plant extracts (Sawong et al., 2022). In recent years, the role of cardenolides as a potential anticancer agent was revealed and has emerged as a possible adjunctive therapy to anticancer therapeutics. Sawong et al. (2022) have shown in a recent study that a combination of low-concentration doxorubicin and calotropin-induced apoptosis is accompanied by suppressed ATP production in HepG2 cells. After this treatment, the production of ATP was reduced, which was associated with the inhibition of glucose intake. Hepatocellular carcinoma cells switch to the glycolysis pathway, but they cannot neutralize ATP deficit so it induced apoptotic cell death in cancer cells. The rate of colorectal cancer is about 4.3% for men and 4.0% for women (https://www.wcrf.org/cancer-trends/colorectal-cancer-statistics/accessed September 2022), so only during 2020, there were more than 1.9 million of new cases (https://www.wcrf.org/cancer-trends/colorectal-cancer-statistics/accessed September 2022) (2022). The combination of low concentrations of 5-FU and calotropin in comparison to a high dose of 5-FU reduced cell viability and abolish resistance to this conventional chemotherapeutic in HCT116 colorectal cancer cells. The same treatment significantly reduced the cellular ATP levels and increase ROS levels in HCT116 cells (Winitchaikul et al., 2021). Additionally, the increased intracellular concentration of ROS triggers autophagy and induced apoptotic cell death suppressing the activity of antioxidative enzymes (Scherz-Shouval et al., 2019). The level of the expression of autophagy-related genes could be reduced or even completely absent in cancer cells after calotropin treatment (Pederson et al., 2020).

## 6 Limitations

The therapeutic limitations and clinical pitfalls of calotropin as an anticancer agent are represented by insufficient data on adverse and toxic effects in humans. Very few cases have reported some digestive, hepatotoxic adverse effects and only one case with severe cardiotoxicity (Iyadurai et al., 2020). Other important limitations are the lack of clinical studies of calotropin in cancer, and the lack of *in vivo* studies of calotropin in experimental animal models for all cancers. As a result, these studies are needed in the future to confirm its anti-cancer therapeutic potential. Translational pharmacological studies are also needed to establish effective therapeutic doses in humans.

## 7 Conclusion

Traditional medicine from the Middle East, India, and China has a long period of usage, development and impact on the later evolvement of a great number of approved drugs and supplements. Until today this traditional medicine has been recognized as one of the most diverse with daily consumption by a huge number of their citizens. Despite their long-term usage, for most of the traditionally used plants, the full mechanism of action has not been discovered yet. However, modern medicine in the last few decades turns to reveal its true impact on health improvement and to confirm its mechanism of action. Finding the critical molecules in these traditionally used plants has a further influence on the development of modern drugs approved by different regulatory bodies across the world. Studies have shown that calotropin has cytotoxic and antitumor effects, the most representative effects being on breast, colon, lung and leukemia cancers. The main limitation of calotropin is represented by the fact its anticancer activity has been confirmed *in vitro*, while there is a lack of *in vivo* evidence, which is especially noted regarding human clinical studies; there are no available clinical trials where calotropin was investigated. Increasing interest in the anticancer effects of calotropin and numerous evidences of its activity on cancer cell lines puts calotropin on the pedestal of cancer research. Additionally, the encouraging evidence that the cytotoxic effects of calotropin can be avoided on normal cell lines makes it a promising agent for further research. There is not a lot of evidence of anticancer activity for chemicals obtained from plant-like there are for calotropin. However, it is evident that *in vivo* experiments on animals are still deficient, and that should be the next step in calotropin research.
