# Supplementary material for: An updated pharmacological insight into calotropin as a potential therapeutic agent in cancer
Source: Front Pharmacol. 2023 Apr 17;14:1160616. doi: 10.3389/fphar.2023.1160616 (PMC10149670; doi:10.3389/fphar.2023.1160616)
Supplement: Supplementary file 1 [file DataSheet1.docx]

**Chemical characterization of calotropin and its derivatives**

The chemical formula of calotropin is C_29_H_40_O_9_. Three components build the structure of the calotropin molecule: a steroid structure of four fused C rings, a butenolide - 5-membered lactone group at C17, and the sugar moiety forming a 1,4-dioxane ring doubly attached to the C3 position of the first carbon ring (Figure 1)(Agrawal et al., 2012).


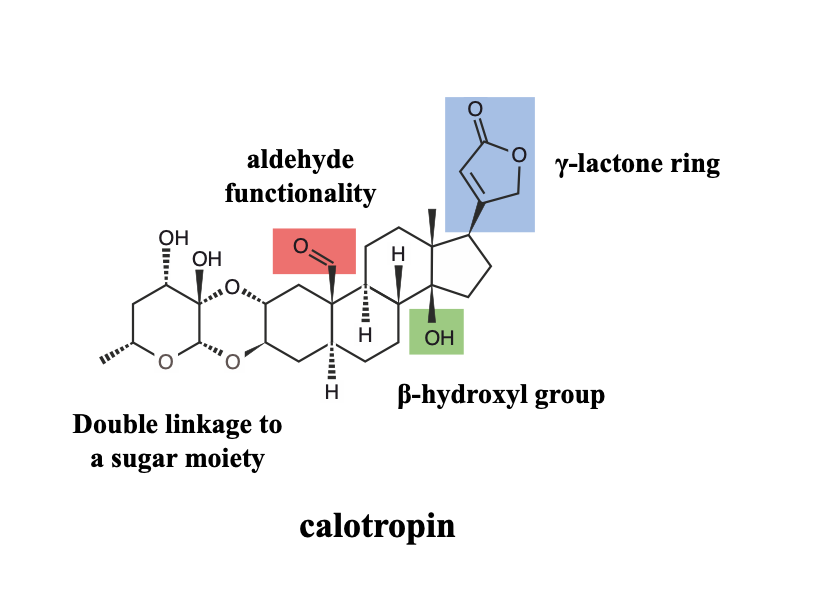


**Figure 1.** Chemical structure of calotropin.


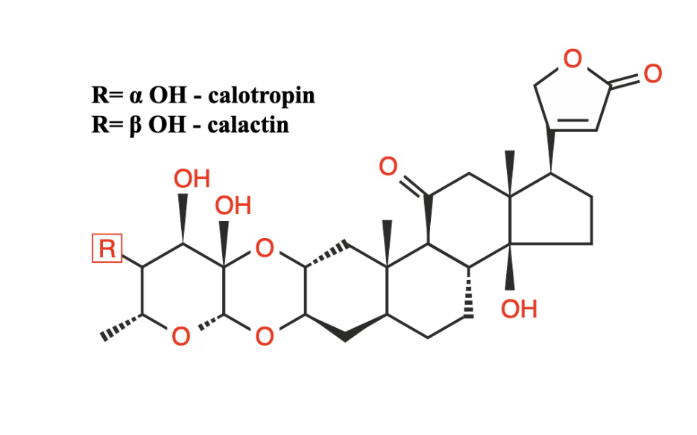


**Figure 2.** The structure of calotropin and its configurational isomer calactin


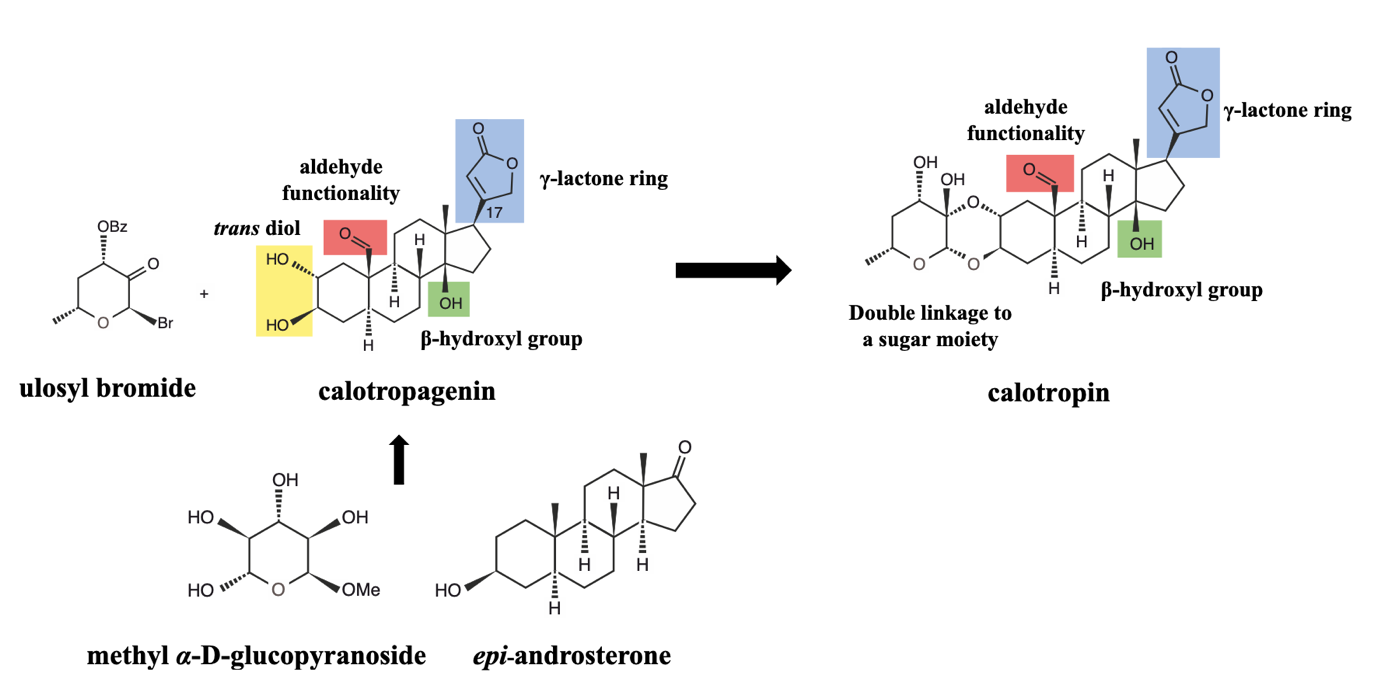
For the synthesis of calotropin like for other cardenolides, the precursor is calotropagenin (Koch et al., 2020). The synthesis of calotropagenin starting from *epi*-androsterone comprises the following aspects: oxidation of C2 and C19 to enable the introduction of the trans 2α,3β-diol structural motif and aldehyde functionality; diastereoselective introduction of the β-hydroxyl group under inversion of the present stereogenic centre; introduction of the γ-butenolide ring at C-17 (Figure 3). Thus, a strategy was developed for calotropin semi-synthesis starting from readily available materials like epi-androsterone and methyl α-D-glucopyranoside.

**Figure 3.** Scheme synthesis of calotropin

Also, calotropagenin is a precursor for calactin, calotoxin, and uscharin (Koch et al., 2020). They are often found together in plants of the family Asclepiadaceae. Jolad et al. (Jolad et al., 1986) isolated calotropin, calactin, uscharidin, uscharin, uzarigenin-3-β-glucoside, and derivatives from the species *Asclepias subulate* (family Asclepiadaceae).


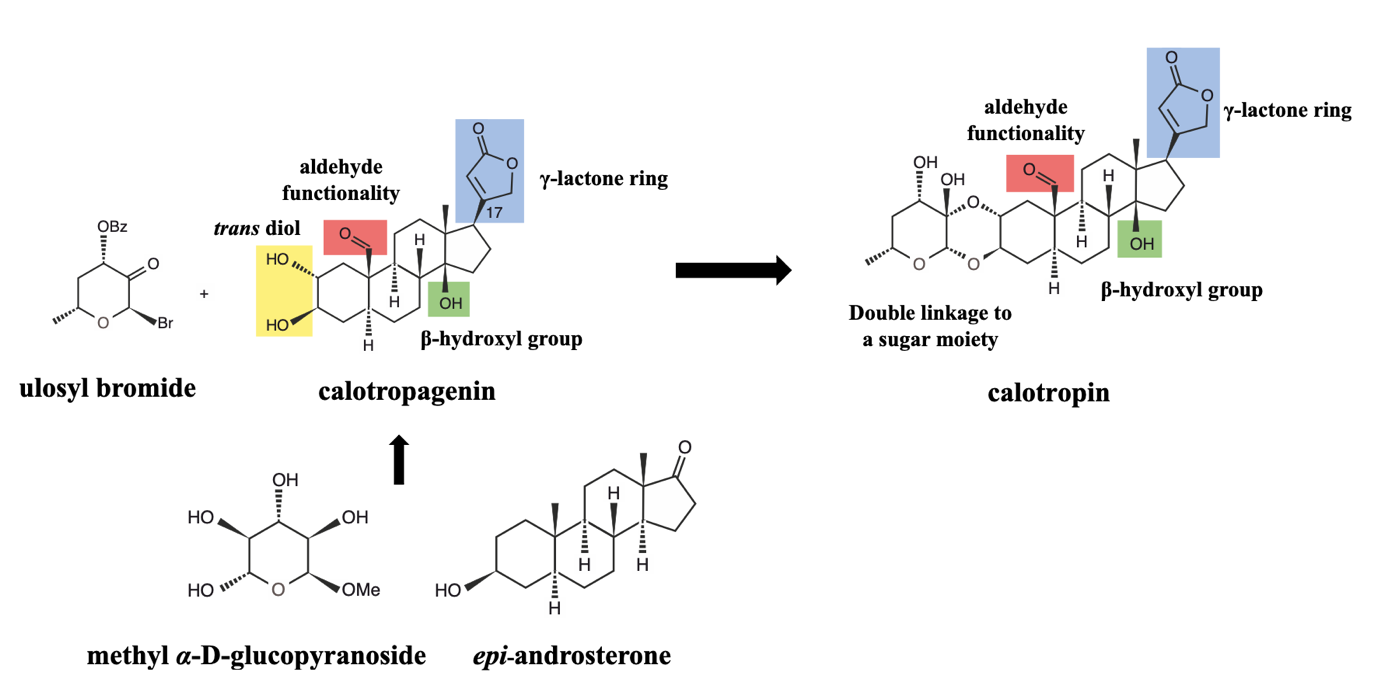
For the synthesis of calotropin like for other cardenolides, the precursor is calotropagenin (Koch et al., 2020). The synthesis of calotropagenin starting from *epi*-androsterone comprises the following aspects: oxidation of C2 and C19 to enable the introduction of the trans 2α,3β-diol structural motif and aldehyde functionality; diastereoselective introduction of the β-hydroxyl group under inversion of the present stereogenic centre; introduction of the γ-butenolide ring at C-17 (Figure 3). Thus, a strategy was developed for calotropin semi-synthesis starting from readily available materials like epi-androsterone and methyl α-D-glucopyranoside.

**Figure 3.** Scheme synthesis of calotropin

Also, calotropagenin is a precursor for calactin, calotoxin, and uscharin (Koch et al., 2020). They are often found together in plants of the family Asclepiadaceae. Jolad et al. (Jolad et al., 1986) isolated calotropin, calactin, uscharidin, uscharin, uzarigenin-3-β-glucoside, and derivatives from the species *Asclepias subulate* (family Asclepiadaceae).

Cardiac glycosides show several identic features, for example, they possess a tertiary hydroxyl group at C14 and a β-oriented butenolide substituent at C17 - 5β cardenolides (Agrawal et al., 2012). They consist of two parts, i.e., an aglycone fragment (non-sugar) and a sugar fragment (Figure 4) (El-Seedi et al., 2019). While digitoxin, digoxin (Figure 5), and ouabain are attached via their 3β-OH group to the sugar moiety, the cardenolides as calotropin (Figure 4) and its related cardenolides - calactin, calotoxin, asclepin, uscharin, uscharidin are connected by the 2α- and 3β-position to the sugar unit forming a 1,4-dioxane ring (Koch et al., 2020). Modifications occur mostly at C3′ and include acetylation (Singh and Rastogi, 1972) or epimerization (Brown et al., 1979) of the hydroxyl group, but also its oxidation (Koch et al., 2020). Moreover, an attachment of a thiazolidine (Singh and Rastogi, 1972) or a dihydro thiazolidine (Koch et al., 2020) moiety was discovered at C3′ as a frequent modification of the sugar building blocks demonstrating the broad diversity of the presented cardenolide class.


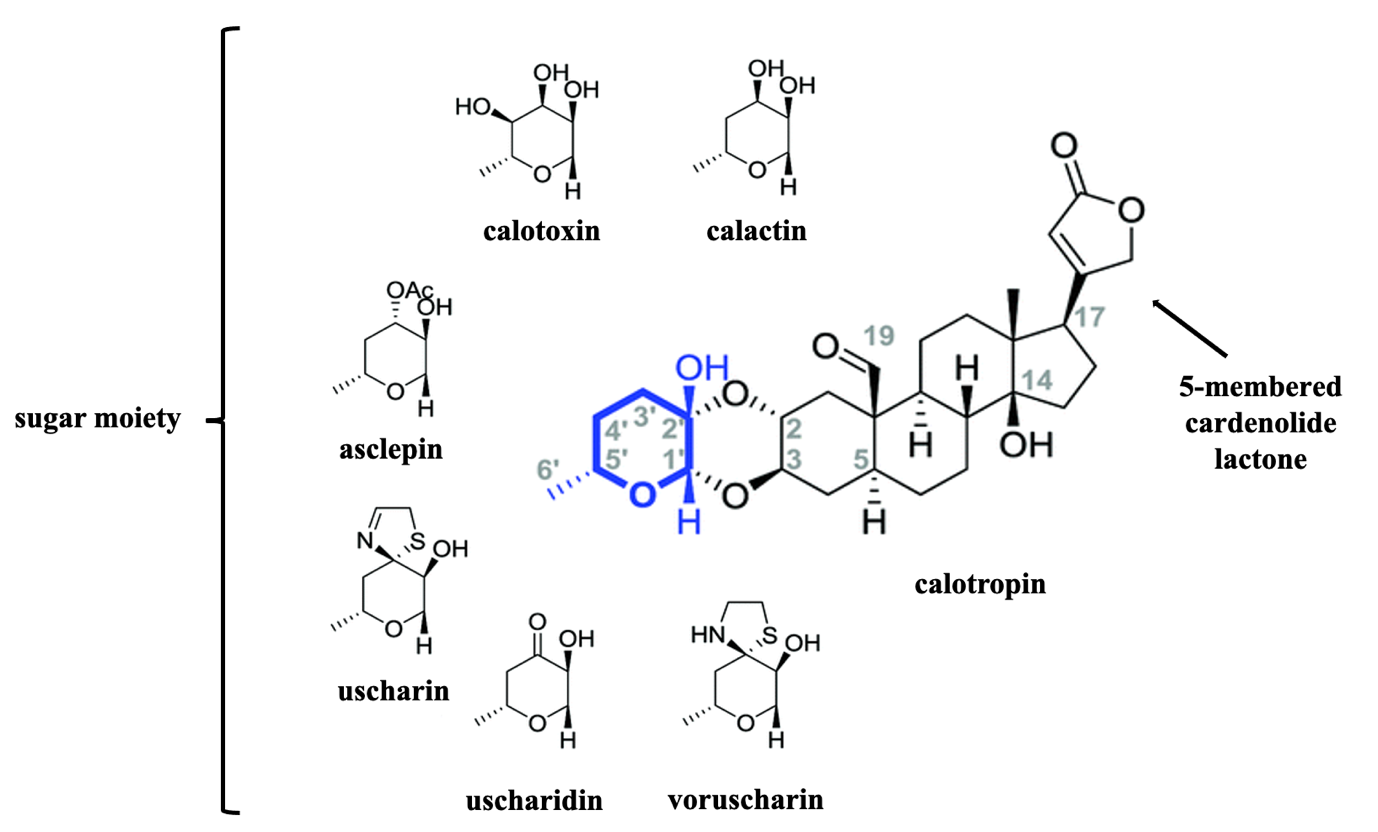


**Figure 4.** The basic skeleton of cardiac glycosides on the example of calotropin and selected cardenolide glycosides isolated from the family *Asclepiadaceae* plants. This figure was adopted from Koch et al (Koch et al., 2020).


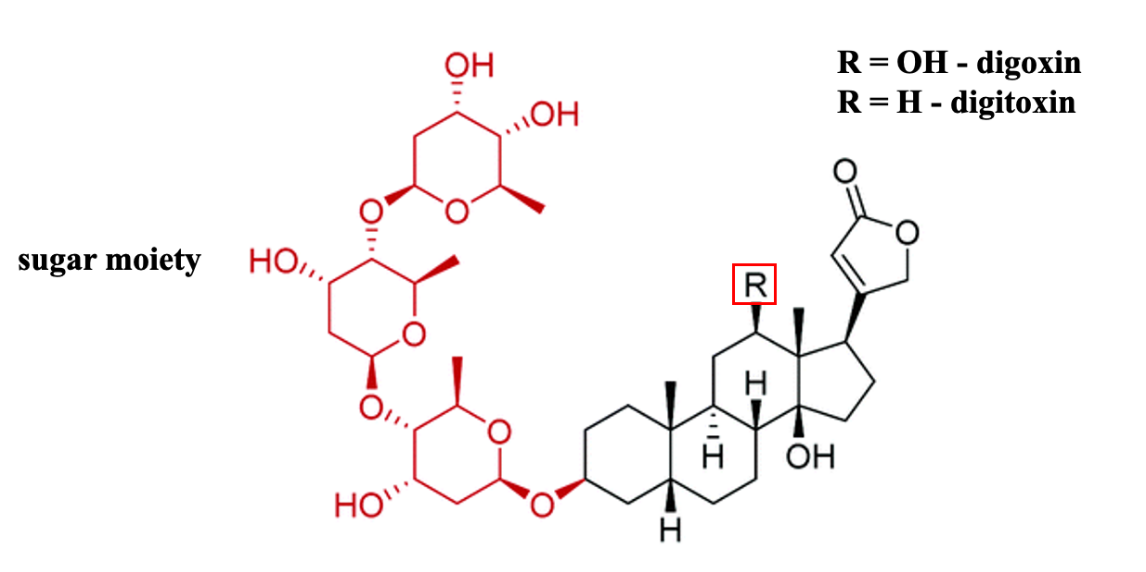


**Figure 5.** The basic skeleton of cardiac glycosides digoxin and digitoxin.

The different substitution patterns at the sugar moiety given a wide range of derivatives (Figure 5). Therefore, as calotropin, they could serve as a lead structure for new therapeutics in the treatment of cancer. Further, the A/B and C/D rings of cardiac glycosides as digoxin and digitoxin, are usually *cis*-fused whereas calotropin and other cardiac glycosides isolated from the family Asclepiadaceae plants have a *trans*-junction of the A/B rings (Figure 6) (Malcolm, 1991).


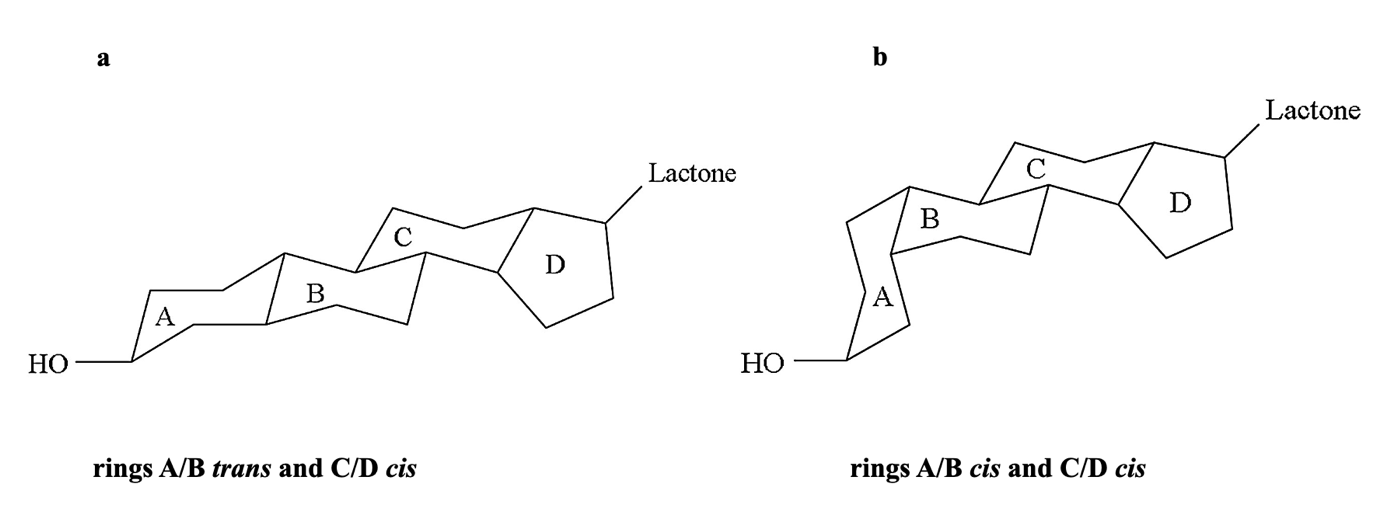


**Figure 6.** The spatial configuration of cardenolides found in plants from the family Asclepiadaceae (a) and spatial configuration of most cardenolides (b)

The therapeutic use of cardiac glycosides for more than two centuries for the treatment of heart failure and as a diuretic has been well documented. However, there is more and more evidence that they can play a significant role in the treatment of various types of cancer. These compounds are considered reliable platforms for the development of many front-line drugs for the treatment of proliferative diseases such as cancer. The minimum structure of cardenolides that is necessary for receptor recognition (and therefore toxicity to animals) is the steroid nucleus 5β,14β-androstane-3β14-diol (Repke, 1985). Lactone rings and sugar moieties are dispensable structures for biological activity (Repke, 1985) but are nonetheless predicted to play a primary role in the interaction with Na+/K+-ATPase (Agrawal et al., 2012, Paula et al., 2005). Previous research has shown that structurally different cardenolides exert quantitatively different toxic effects (Agrawal et al., 2012). The configuration of the A/B and C/D ring junctions (Figure 6) is important for toxicity. Whereas the cis configuration at the C/D ring junction is mandatory, the cis configuration at both junctions renders cardenolides the most toxic (Agrawal et al., 2012). Calotropin and other cardenolides which possess a trans configuration at the A/B junction have weaker toxicity (Hoch, 1961). In addition, cardenolides which have one sugar are usually more active than cardenolides with two sugars. Hoch and colleagues (Hoch, 1961) have shown that successive removal of terminal sugar from cardenolides with bi- or trio-side sugar can lead to a stepwise increase in toxicity (at least in mammals). It was previously concluded that the removal of sugar might represent a detoxification mechanism in the caterpillar of the monarch butterfly (Seiber et al., 1983).

**References**

AGRAWAL, A. A., PETSCHENKA, G., BINGHAM, R. A., WEBER, M. G. & RASMANN, S. 2012. Toxic cardenolides: chemical ecology and coevolution of specialized plant-herbivore interactions. *New Phytol,* 194**,** 28-45.

BROWN, P., VON EUW, J., REICHSTEIN, T., STÖCKEL, K. & WATSON, T. R. 1979. Cardenolides of Asclepias syriacaL., Probable Structure of Syrioside and Syriobioside. Glycosides and aglycones, 334th communication. 62**,** 412-441.

EL-SEEDI, H. R., KHALIFA, S. A. M., TAHER, E. A., FARAG, M. A., SAEED, A., GAMAL, M., HEGAZY, M. F., YOUSSEF, D., MUSHARRAF, S. G., ALAJLANI, M. M., XIAO, J. & EFFERTH, T. 2019. Cardenolides: Insights from chemical structure and pharmacological utility. *Pharmacol Res,* 141**,** 123-175.

HOCH, J. H. 1961. *A survey of cardiac glycosides and genins*, University of South Carolina Press.

JOLAD, S. D., BATES, R. B., COLE, J. R., HOFFMANN, J. J., SIAHAAN, T. J. & TIMMERMANN, B. N. 1986. Cardenolides and a lignan from asclepias subulata. *Phytochemistry,* 25**,** 2581-2590.

KOCH, V., NIEGER, M. & BRÄSE, S. 2020. Towards the synthesis of calotropin and related cardenolides from 3-epiandrosterone: A-ring related modifications. *Organic Chemistry Frontiers,* 7**,** 2670-2681.

MALCOLM, S. B. 1991. Chapter 7 - Cardenolide-Mediated Interactions between Plants and Herbivores. *In:* ROSENTHAL, G. A. & BERENBAUM, M. R. (eds.) *Herbivores: their Interactions with Secondary Plant Metabolites (Second Edition).* San Diego: Academic Press.

PAULA, S., TABET, M. R. & BALL, W. J. J. B. 2005. Interactions between cardiac glycosides and sodium/potassium-ATPase: three-dimensional structure− activity relationship models for ligand binding to the E2-pi form of the enzyme versus activity inhibition. 44**,** 498-510.

REPKE, K. J. T. I. P. S. 1985. New developments in cardiac glycoside structure-activity relationships. 6**,** 275-278.

SEIBER, J., LEE, S. & BENSON, J. J. M. D., AMSTERDAM 1983. Handbook of Natural Toxins. 1**,** 43-83.

SINGH, B. & RASTOGI, R. P. 1972. Structure of asclepin and some observations on the NMR spectra of Calotropis glycosides. *Phytochemistry,* 11**,** 757-762.
